# Research on MOOCs course recommendation system based on hybrid attention mechanism in frequency and time domain

**DOI:** 10.1371/journal.pone.0338738

**Published:** 2025-12-30

**Authors:** Hongli Yuan, Li Liu, Yiwen Zhang, Guangwei Wang, Aixiang He, Fucheng Zhang

**Affiliations:** Big Data and Artificial Intelligence College, Anhui Xinhua University, Hefei, China; Sreenidhi Institute of Science and Technology, INDIA

## Abstract

Course recommendation systems serve as a critical component of online education platforms, playing a vital role in enhancing learning efficiency and personalized experiences. However, existing recommendation approaches, including recent sequential models such as BERT4Rec and LightSANs, primarily concentrate on temporal-domain modeling of user behaviors while neglecting the potential of frequency-domain analysis. This leads to incomplete characterization of user behavior patterns, particularly presenting challenges in capturing stable long-term interests from sparse and noisy interaction data. To address these limitations, this study proposes a novel hybrid attention network for Massive Open Online Courses Course recommendation, designed to jointly model both frequency-domain and temporal-domain features. The model employs Fast Fourier Transform to extract frequency-domain characteristics from user behavior sequences while utilizing a self-attention mechanism to capture temporal dynamics, thereby enabling collaborative modeling of dual-domain features. Experimental results on the public MooCCube dataset demonstrate that the proposed model achieves Hit Ratio@10, MRR@10, and NDCG@10 scores of 0.4534, 0.2018, and 0.2618, respectively, outperforming current mainstream recommendation algorithms. Ablation studies further validate the effectiveness of dual-domain fusion, with approximately 10% and 5% performance improvements in NDCG@10 and Hit@10 compared to single-domain approaches. This research provides a novel technical pathway for overcoming performance bottlenecks in personalized course recommendation.

## Introduction

Against the backdrop of rapid digital education evolution, Massive Open Online Courses (MOOCs) have become a central pillar of modern lifelong learning systems, providing global learners with unprecedented access to high-quality, interdisciplinary educational resources [1]. Leading MOOC platforms like XuetangX and Coursera now host tens of thousands of courses, serving the diverse learning needs of millions of users worldwide [2]. However, the exponential growth of course resources has also triggered an “information overload” dilemma—learners often struggle to pinpoint resources that precisely align with their academic goals, prior knowledge, and learning pace within vast course libraries [3]. Unlike e-commerce recommendation scenarios, MOOC course selection is constrained by two core factors: First, structural dependencies within course systems (e.g., “Advanced Mathematics” as a prerequisite for “Machine Learning”) [4,5]. Second, long-term learning cycles (e.g., semester-based phased study plans) [6]. These unique attributes impose higher demands on recommendation models, necessitating the development of technical frameworks capable of simultaneously capturing both the dynamic evolution of user interests and the inherent logic of educational content.

Existing course recommendation systems can be broadly categorized into two approaches: non-temporal and temporal recommendation methods. Non-temporal methods are designed based on the assumptions that “user preferences remain static and independent across courses.” However, the MOOC learning process inherently involves the coupling of “dynamic preferences with a structured course system.” This fundamental mismatch renders non-temporal methods fundamentally unsuitable for MOOC scenarios. To address this limitation, temporal recommendation methods have emerged as a research focus, with the core objective of enhancing recommendation accuracy by modeling the temporal dependencies in user behavior. Early temporal models, exemplified by the Factorized Personalized Markov Chain (FPMC), combined matrix factorization with Markov chains to capture short-term sequence patterns [7,8]. However, these traditional statistical approaches struggle to handle complex nonlinear relationships within long sequences. The advent of deep learning technologies revolutionized temporal recommendation: Recurrent Neural Networks (RNNs) and their variants (LSTMs, GRUs) effectively model long-term temporal dependencies; Convolutional Neural Networks (CNNs) (e.g., the Caser model) excel at extracting local sequence features; while Transformer-based architectures, leveraging self-attention mechanisms for parallel modeling of global context, have become the mainstream solution. Models such as CORE [9], SINE [10], LightSANs [11], and BERT4Rec [12] have demonstrated outstanding performance in sequence recommendation tasks.

Although the aforementioned models have achieved breakthroughs in the field of temporal recommendation, they all share a critical limitation—overreliance on single-dimensional temporal domain analysis, neglecting the value of frequency domain patterns inherent in user learning behavior. User learning behavior commonly exhibits periodic characteristics, such as participating in programming courses during fixed weekly time slots or systematically revisiting core statistics modules at the start of each semester. These patterns, reflecting learners’ deep-seated study habits, cannot be fully captured by temporal models alone. Recent research has begun exploring frequency domain analysis in recommendation systems. Du et al. [13] proposed a frequency-enhanced hybrid attention network that extracts periodic features from sequences via Fast Fourier Transform (FFT), demonstrating that frequency domain information effectively complements temporal dynamic features. However, the application of such frequency-time hybrid models in MOOC scenarios remains unexplored, particularly lacking tailored solutions for addressing MOOC-specific constraints such as course dependencies and extended learning cycles.

In response to the aforementioned challenges, the main contributions of this study can be summarized as follows:

(1) We propose a hybrid attention network incorporating frequency domain enhancement for course recommendation. This study introduces frequency domain analysis into MOOCs recommendation tasks. The proposed model jointly utilizes the Fast Fourier Transform to capture global frequency domain patterns and employs a self-attention mechanism to model local temporal dynamics, thereby achieving a more comprehensive representation of user behavior.

(2) Comprehensive experimental validation was conducted on publicly available benchmark datasets. Extensive experiments on the public MOOCCube dataset demonstrate superior performance, achieving Hit Ratio@10, MRR@10, and NDCG@10 scores of 0.4534, 0.2018, and 0.2618, respectively. These results confirm the model’s superiority over multiple strong baseline methods in both recommendation accuracy and generalization capability.

(3) Conducted in-depth analysis and elucidated broader application value. Through rigorous ablation experiments, we quantitatively validated the necessity of fusing frequency and time domains: compared to single-domain approaches, performance improvements of approximately 10% and 5% were achieved for NDCG@10 and Hit@10 metrics, respectively. This study not only provides a novel modeling perspective for educational recommendation systems but also offers a transferable solution framework for other sequence recommendation tasks.

## Related research

The evolution of sequence recommendation systems has long been constrained by the temporal modeling paradigm, which struggles to capture the inherent cyclical learning behaviors in massive open online courses (MOOCs). This section deconstructs the developmental trajectory of sequence models—from traditional statistical approaches to modern neural architectures—focusing on the technical characteristics and limitations of mainstream temporal models. It reveals a fundamental and long-overlooked research gap: the absence of frequency-domain analysis.

### Statistical sequence models

Early research, exemplified by Factorized Personalized Markov Chains (FPMC) [8], employed Markov chains to capture linear transition patterns between sequential behaviors. While these models laid the foundation for sequence recommendation, they suffer from a fundamental flaw: an inability to model nonlinear, long-range dependencies. In the MOOC context, this limitation is particularly pronounced due to the complex multi-step prerequisite chains between courses (e.g., “Calculus → Linear Algebra → Machine Learning”). More critically, such models completely lack the ability to recognize periodic patterns, failing to detect regular learner behaviors such as “weekly quiz participation” or “semester-based course planning” [7]. This results in recommendations that are severely misaligned with learners’ academic rhythms.

### Deep learning-based sequence models

The advent of deep learning has offered hope for overcoming the limitations of traditional statistical sequence models. Various neural architectures have driven breakthrough improvements in temporal recommendation performance through their nonlinear modeling capabilities and efficient feature extraction mechanisms. Models such as CORE [9], SINE [10], and LightSANs [11] have formed differentiated technical approaches tailored to specific scenarios, emerging as the current mainstream comparison solutions. RNN/LSTM-based models (e.g., GRU4Rec [14]) pioneered nonlinear state transition mechanisms. By dynamically adjusting hidden layer information through gating units, they effectively capture temporal dependencies in user behavior sequences, demonstrating foundational efficacy in MOOC session-level recommendations. However, sequential computation leads to inefficient processing of long sequences, and the “vanishing gradient” makes it difficult to associate logically connected course selections at a distance (e.g., the prerequisite relationship between “Advanced Mathematics” and “Machine Learning” taken six months later). CNN-based models (e.g., Caser [15]) leverage parallel computation through convolutional filtering, excelling at extracting local enrollment patterns (e.g., sequential associations like “Python Fundamentals → Advanced SQL”). However, their limited receptive field prevents modeling global periodic behaviors, and they overlook course knowledge structure dependencies, resulting in insufficient recommendation coherence.

Self-attention models (such as SASRec [12] and BERT4Rec [16]) represent the current state-of-the-art, leveraging the Transformer architecture to capture global dependencies. BERT4Rec’s bidirectional self-attention and masking mechanism further unearths cross-temporal preference correlations. LightSANs [11] addresses the quadratic complexity issue of traditional self-attention by proposing a low-rank decomposition self-attention mechanism. Combined with decoupled location encoding to separate item and location relevance, it enhances efficiency while mitigating over-parameterization. SINE [10] focuses on the challenge of dispersed user interests, optimizing recommendation diversity through its "Interest Activation - intent aggregation" framework to optimize recommendation diversity: pre-constructing a large orthogonal interest concept pool while resolving the consistency issue between training and inference in traditional multi-interest models. CORE [9] addresses feature heterogeneity by mapping user session behaviors and course attributes to a unified representation space via multilayer perceptrons, introducing contrastive learning to enhance feature consistency. This effectively combines recent behaviors with course attributes in short-term session recommendations. However, none of these models transcend the temporal domain. Research by W. Song et al. [17] reaffirms that such single-temporal-domain models exhibit significant performance deficiencies when predicting non-continuous learning requests (typically periodic patterns), highlighting structural limitations.

### Graph neural networks and large language models

Recent research has increasingly emphasized the role of external knowledge structures and semantic understanding techniques in recommendation systems to address the limitations of traditional behavioral sequence data. In modeling structured knowledge, G. Zhang et al. [18] employed graph convolutional networks to jointly model complex inter-course relationships and user learning styles, effectively capturing knowledge dependency characteristics in MOOC scenarios. To address data sparsity, particularly in cold-start scenarios, F. Huang et al. [19] innovatively employed large language models to generate synthetic user behavior sequences. This semantic-level data augmentation significantly improved model performance on sparse datasets. Concurrently, multimodal information fusion [20] and semi-supervised learning methods [21] have been widely applied to extract deeper user cognitive states from multi-source data such as course discussions.

While these approaches enrich feature representations by incorporating knowledge graphs and textual semantics, they fundamentally remain supplementary enhancements to core sequence modeling. Ultimately, this external information must still be processed through time-domain-based sequence recommendation frameworks (e.g., Transformers or RNNs), failing to revolutionize the underlying mechanism of sequence modeling—namely, transitioning from singular time-domain analysis to collaborative time-domain and frequency-domain modeling. Consequently, while existing methods improve recommendation effectiveness, they have not overcome the inherent limitations of time-domain modeling and remain unable to effectively capture the intrinsic periodic patterns of user behavior in MOOC scenarios.

The frequency-enhanced hybrid attention network proposed by Du et al. [13] represents a pivotal turning point in the field of frequency-aware recommendation systems. This model leverages the Fast Fourier Transform (FFT) to uncover latent periodic patterns within user behavior. However, its design and evaluation are tailored for short-term, unstructured domains such as e-commerce, failing to adequately account for the long-term, macro-scale cycles inherent in the MOOC ecosystem—such as weekly or semester-based learning cycles—and the course structures constrained by strict prerequisite knowledge requirements.

## Methodology

**Fig 1:**
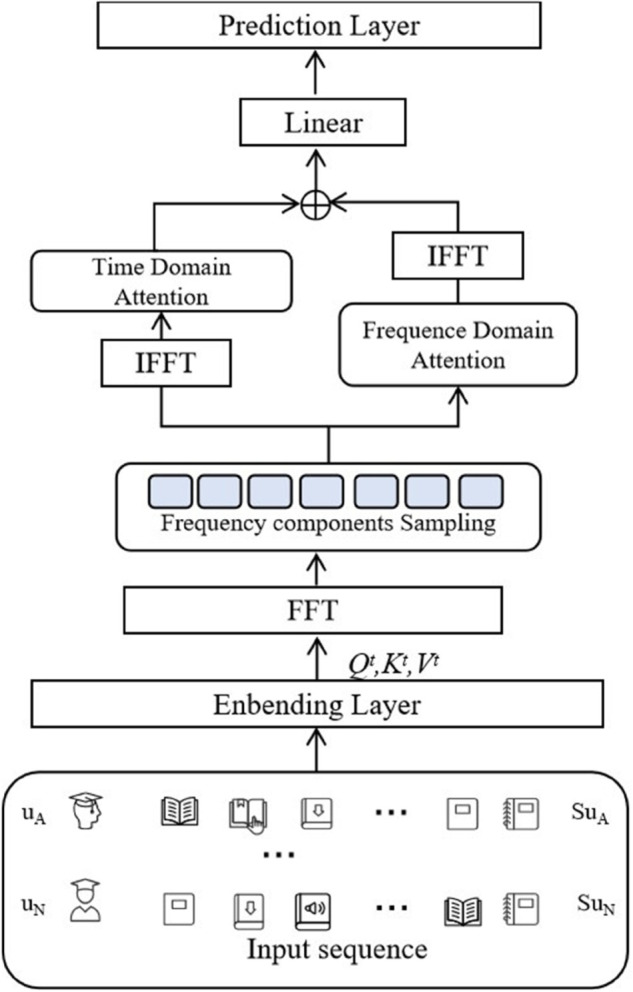
Frequency-time hybrid attention model.

In this study, we propose a MOOC course recommendation system called Frequency-Time Hybrid Attention Model. Frequency-Time Hybrid Attention Model based on a hybrid attention mechanism in both frequency and time domains, which improves the accuracy of personalized course recommendation by jointly modeling the dynamic changes in the time domain and the periodic laws in the frequency domain of user behavior data. The overall architecture of the model and the design of each module are described in Fig 1.

The overall architecture of the proposeed model consists of the following modules: Enbending Layer, Time-domain attention module, Frequency-domain attention module and recommendation prediction layer. These modules work closely with each other to gradually realize feature extraction, time-frequency domain modeling, information fusion and final recommendation generation.

### Time domain attention module

The main task of this module is to model the user’s time-series behavioral characteristics and capture the explicit and implicit dynamic preference information in the interaction sequence. Through the Self-Attention mechanism (Self-Attention), it can effectively mine the short-term interest, long-term dependency and global context information in the sequence. This is the basic module for modeling user personalized preferences in recommender systems.

- **Representation of the input sequence**

The interaction history of learnercan be represented as an ordered sequence of courses $S_{u}=[c_{1},c_{2},\ldots ,c_{i},\ldots ,c_{n}]$, where *c*_*i*_ is the user’s interaction behavior with course. Each course will be mapped to *d*-dimensional embedding vector space, as in (1).

$$E=[e_{1},e_{2},\ldots ,e_{i},\ldots ,e_{n}],e_{i}\in {\mathbb{R}}^{d}.$$ (1)

where *E* is the course embedding matrix containing the embedding vector for each course in the interaction history.

- **Positional Encoding**

The self-attention mechanism is inherently disordered, so Positional Encoding (Positional Encoding) needs to be introduced to preserve the order information of the sequence. Positional Encoding can be calculated by the following Formula (2).

$$P_{i,2k}=\sin\left(\frac{i}{10000^{\frac{2k}{d}}}\right),\;P_{i,2k+1}=\cos\left(\frac{i}{10000^{\frac{2k}{d}}}\right).$$ (2)

where *i* denotes the position in the sequence and k denotes the dimension index. Eventually, the course embedding vectors are summed with the position encoding (3).

E=E+P. (3)

- **Self-attention mechanism computation**

The self-attention mechanism generates a weighted time series representation by computing the correlation of each pair of course embeddings in the sequence. The input embedding matrix $E\in {\mathbb{R}}^{n\times d}$ is mapped to the Query, Key, and Value vector space, as in (4).

$$Q=EW_{Q},K=EW_{K},V=EW_{V}.$$ (4)

where $W_{Q},W_{K},W_{V}\in {\mathbb{R}}^{n\times d}$is the learnable weight matrix.

*d*_*k*_ is the dimension of the query and key vectors (usually set to *d*_*k*_ = *d*). The dot product of the query and key vectors is used to measure the correlation between courses at different locations to generate the attention weight matrix (5).

$$A=\text{softmax}\left(\frac{QK^{𝖳}}{\sqrt{d_{k}}}\right).$$ (5)

where $A\in {\mathbb{R}}^{n\times n}$ is the attention weight matrix and *A*_*i*,*j*_ denotes the attention weight of the ith course to the jth course. $\sqrt{d_{k}}$ is the scaling factor, which is used to mitigate the problem of excessively large dot product values.

A weighted representation of the sequence is obtained by weighting the value vector V using the attention weights.

$$H_{T}=AV.$$ (6)

where $H_{T}\in {\mathbb{R}}^{n\times dk}$ is the time-domain feature matrix. Each row corresponds to the time-domain contextual representation of each course in the user sequence.

To enhance the expressive power of the model, the self-attention mechanism is often extended to Multi-Head Attention. Diverse relationships of sequences are captured through multi-group queries, key and value vector computations.

$$MultiHead(Q,K,V)=Concat(head_{1},\ldots ,head_{i},\ldots ,head_{h})W_{O}.$$ (7)

Where: ${\text{head}}_{i}=\text{Attention}\left(QW_{Q}^{\left(i\right)},KW_{K}^{\left(i\right)},VW_{V}^{\left(i\right)}\right)$. *h* is the number of heads, $W_{Q}^{\left(i\right)},\;W_{K}^{\left(i\right)},\;W_{V}^{\left(i\right)}$ is the projection matrix of the *i*th head. $W_{O}\in {\mathbb{R}}^{hdk\times d}$ is the output matrix.

- **Residual linking and normalization**

To avoid gradient vanishing and to enhance the stability of the model, residual linking with layer normalization is used.

$$H_{T}=\text{LayerNorm}\left(H_{T}+E\right).$$ (8)

The final generated time-domain feature *H*_*T*_ provides a time-dynamic representation of user behavior, which is able to capture both short-term changes and long-term stability of user interests, and provides a rich feature representation for downstream tasks.

### Frequency domain modeling module

The frequency domain modeling module aims at extracting periodic features from sequences of learner behaviors in order to reveal deep patterns hidden in temporal variations. For example, a learner may regularly participate in a certain type of class (e.g., a weekly programming class) during a specific period of time, and these patterns are difficult to capture directly through the time domain. By transforming the time series to the frequency domain through Fast Fourier Transform (FFT), it is possible to identify learners as significant at specific frequencies.

- **Representation of the input sequence**

The input data is a sequence of user interaction history behaviors $S_{u}=[C_{1},C_{2},\ldots ,C_{i},\ldots ,C_{n}]$, where *C*_*i*_ denotes the learner’s interactions with course i and each interaction is represented as a *d*-dimensional embedding vector. Therefore, the user’s interaction sequence can be represented as an embedding matrix $E=[e_{1},e_{2},\ldots ,e_{i},\ldots ,e_{n}]$, where each $e_{i}\in {\mathbb{R}}^{d}$ is an embedding vector of the course.

- **Frequency domain feature extraction (Fast Fourier Transform FFT)**

In order to capture the periodic features in the learner behavior data, it is first necessary to convert the time-series data to the frequency domain. Suppose the input learner behavior data is $X=[x_{1},x_{2},\ldots ,x_{i},\ldots ,x_{n}]$, which *x*_(*i*)_ represents the *i* moment interaction features or embedded features. Fast Fourier Transform (FFT) is applied to convert it to frequency domain.

$$X_{f}=FFT(x).$$ (9)

The Fourier transform results *X*_*f*_ in a frequency domain component in complex form which can be expressed as in (10).

$$X_{f}=A_{f}+jB_{f}.$$ (10)

where $A_{f}\in {\mathbb{R}}^{n}$ is the magnitude information in the frequency domain and $B_{f}\in {\mathbb{R}}^{n}$ is the phase information in the frequency domain. The magnitude information *A*_*f*_ in the frequency domain describes the strength of the individual frequency domain into phase in the signal, while the phase information *B*_*f*_ indicates the time offset of the individual frequency components.

- **Frequency domain magnitude information extraction**

In the course recommendation task, the magnitude information is usually more recognizable than the phase information, therefore, this paper focuses on the magnitude component *A*_*f*_ in the frequency domain. To extract this information, the magnitude of each frequency component can be modeled. Specifically, by calculating the magnitude of each frequency component.

$$A_{f}=|X_{f}|.$$ (11)

The amplitude vector *A*_*f*_ is then modeled using the amplitude vector as a frequency domain feature.

- **Frequency domain attention mechanism**

In order to further highlight the frequency components that are important for the recommendation task, the frequency domain attention mechanism is introduced. This mechanism helps the model to focus on important frequency components by adaptively assigning weights to different frequency components. Assuming that the frequency domain feature *A*_*f*_, is a vector of length *n*, the frequency domain attention mechanism can be computed by the following steps.

First, the attention weights are computed by inputting the magnitude information *A*_*f*_ into a fully connected layer or linear mapping to generate frequency domain attention weights.

$$A_{f}^{\prime }=A_{f}W_{f}.$$ (12)

where $W_{f}\in {\mathbb{R}}^{1\times df}$ is the weight matrix and $A_{f}^{\prime }$ is the mapped feature vector. Then, this feature vector is converted to a probability distribution by Softmax operation to obtain the frequency domain attention weights.

$$\alpha_{f}=\text{softmax}\left(A_{f}^{\prime }\right).$$ (13)

where $\alpha_{f}\in {\mathbb{R}}^{n}$ is the attentional weight of each frequency component, indicating the importance of that frequency component.

Next, the frequency domain magnitude vectors are weighted by the frequency domain attention weights to obtain a weighted frequency domain feature representation.

$$H_{F}=\alpha_{f}⊙A_{f}.$$ (14)

Where, ⊙ denotes element-by-element multiplication. *H*_*F*_ is the frequency domain feature weighted by frequency domain attention.

- **Frequency domain feature reconstruction**

Since the frequency domain features only represent the periodic part of the signal, they usually need to be converted back to the time domain for subsequent processing. The frequency domain feature *H*_*F*_ can be converted back to the time domain by the inverse Fourier transform (IFFT) to obtain the time domain reconstructed feature *X*_*F*_.

$$X_{F}=IFFT(H_{F}).$$ (15)

However, not only can frequency-domain features be used directly in time-domain reconstruction, they actually provide periodicity information in their own right, which can be important in modeling user interest.

- **Output of the frequency domain enhancement module**

The final output of the frequency domain enhancement module is the weighted frequency domain features *H*_*F*_, which provide information about the periodicity and frequency components of user behavior, complementing the time domain features. These frequency-domain features can help capture the cyclical patterns of users’ long-term behaviors and provide an additional dimension of information for subsequent time-series recommendations.

### Hybrid attention mechanism

The hybrid attention mechanism is the core module of the whole model, which is responsible for jointly modeling the time-domain features *H*_*T*_ and the frequency-domain features *H*_*F*_, and fully exploiting the complementation between them in order to generate a multidimensional integrated representation of the user’s behaviors *H*.

$$H=\gamma H_{T}+(1-\gamma )H_{F}.$$ (16)

Where, γ∈[0,1], γ is adaptively adjusted based on the training data to ensure that the fused features are best adapted to the preference patterns of a particular user.

### Recommendation prediction layer loss function and optimization

The goal of a recommender system is to generate a personalized list of recommendations, optimizing the model’s sorting performance and prediction accuracy. When optimizing for a classification task (e.g., whether to hit a course or not), the cross-entropy loss is an effective measure of the difference between the predicted distribution and the true distribution of.

$$L_{\text{CE}}=-\sum_{(u,i)\in D}\left(y_{u,i}\ln({\hat{y}}_{u,i})+(1-y_{u,i})\ln(1-{\hat{y}}_{u,i})\right).$$ (17)

Where *y*_*u*,*i*_ is the actual label and ${\hat{y}}_{u,i}$ is the probability predicted by the model. The Adam optimizer is used to update the gradient of the model parameters. Adam adjusts the strategy with an adaptive learning rate to ensure the efficiency and stability of the training process. The optimization steps are as follows. Update the parameters according to the gradient calculation. A regularization term (e.g. L2 regularization) is used to mitigate the risk of over fitting and optimize the objective function.

$$L=L_{\text{BPR}}+\lambda \|\Theta {\|}^{2}.$$ (18)

Where, in is the regularization coefficient and Θ is all the parameters of the model. Through the above loss function and optimization method, the FTHAN model can effectively learn the complex patterns of user behavior and achieve efficient personalized recommendation.

## Experimental component

### Experimental setup

- **Data set**

This study conducted experiments on the following three publicly available datasets. The statistics of data set information see as Table 1.

**Table 1:** Statistics of data set information.

Dataset	#Users	#Items	#Interaction	Average actions of users	Sparsity
MOOCCube	82,536	1,303	458,453	5.5	99.57%
ml-100K	944	1,683	100,000	106	93.70%
Amazon_Books	86,894	412	100,000	1.2	99.72%

MOOCCube [22]: this dataset is designed for online education recommendation tasks and contains user learning behavior data from the large-scale catechism platform Xue Tang Online. The data includes the click records, learning duration, and learning paths of the users’ learning courses, and is suitable for verifying the effectiveness of the model in educational recommendation scenarios.

MovieLens-100k (ml-100k) [23]: a classic movie recommendation dataset containing 100,000 user ratings of movies from 944 users for 1683 movies. This dataset is widely used for the validation of recommendation algorithms and is characterized by a high density of user-item interactions.

Amazon_Books [24]: user purchase and rating data extracted from Amazon’s book categories, containing rich sequences of user behaviors, which can be used to test the performance of the model in long sequence recommendation tasks.

As shown in Table 1, the publicly available datasets used in this study (e.g., MOOCCube) all exhibit highly sparse characteristics, with sparsity rates exceeding 99.57%. This extreme sparsity represents a common challenge in the field of recommendation systems, posing significant difficulties for models to effectively learn user preferences from limited interactions.

- **Evaluation metrics**

To comprehensively evaluate the performance of the recommender system, this study employs five widely adopted evaluation metrics: Recall@*K*, Precision@*K*, Hit@*K*, MRR@*K*, and NDCG@*K*. These metrics quantitatively assess the recommendation results from various dimensions including coverage, accuracy, ranking quality, and overall utility.

**(1) Recall@*K*** measures the proportion of relevant items in the test set that are successfully recommended, reflecting the system’s ability to cover user interests:

$$\text{Recall@}K=\frac{\sum_{u=1}^{N}|R_{u}(K)\cap T_{u}|}{\sum_{u=1}^{N}|T_{u}|}$$ (19)

where *R*_*u*_(*K*) represents the top-*K* recommended items for user *u*, and *T*_*u*_ denotes the set of true relevant items for user *u* in the test set.

**(2) Precision@*K*** evaluates the accuracy of recommendations by calculating the proportion of relevant items among the top-*K* recommendations:

$$\text{Precision@}K=\frac{\sum_{u=1}^{N}|R_{u}(K)\cap T_{u}|}{N\times K}$$ (20)

**(3) Hit@*K*** serves as a coverage indicator, examining whether at least one relevant item appears in the recommendation list:

$$\text{Hit@}K=\frac{1}{N}\sum_{u=1}^{N}𝕀(|R_{u}(K)\cap T_{u}|>0)$$ (21)

where 𝕀(·) is the indicator function that returns 1 when the condition is true and 0 otherwise.

**(4) MRR@*K*** (Mean Reciprocal Rank) assesses the ranking quality of the recommendation system by calculating the average reciprocal rank of the first relevant item:

$$\text{MRR@}K=\frac{1}{N}\sum_{u=1}^{N}\frac{1}{\min({\text{rank}}_{u},K+1)}$$ (22)

where ${\text{rank}}_{u}$ indicates the rank position of the first relevant item for user *u* in the recommendation list, and when no relevant item is present, the rank is set to *K* + 1.

**(5) NDCG@*K*** (Normalized Discounted Cumulative Gain) comprehensively evaluates the overall utility of recommendation lists by considering both the ranking order and relevance scores:

$$\text{DCG@}K=\sum_{i=1}^{K}\frac{2^{rel_{i}}-1}{\log_{2}(i+1)}$$ (23)

$$\text{NDCG@}K=\frac{1}{N}\sum_{u=1}^{N}\frac{\text{DCG@}K_{u}}{\text{IDCG@}K_{u}}$$ (24)

where *rel*_*i*_ represents the relevance score of the item at position *i*, and $\text{IDCG@}K_{u}$ is the ideal DCG value for user *u* (calculated by sorting all items according to their true relevance in descending order).

All experiments in this study are conducted with *K* = 10, consistent with the common evaluation standards in the recommender systems literature.

- **Baseline model**

In order to validate the effectiveness of the models proposed in this study, four representative models are selected as Baseline for comparison, CORE [9] is a sequence recommendation model, which designs Representation - Consistent Encoder (RCE) to encode session embeddings as linear combinations of item embeddings within a session LightSANs [11] introduce a low-rank decomposition self-attention mechanism, which projects a user’s historical items into a small number of fixed latent interests and utilizes item-interest interactions to generate context-aware representations. SINE [10] proposes a novel sparse interest network for sequence recommendation, where the sparse interest module is able to adaptively generate a context-aware representation for each user from a large pool of concepts, and the sparse interest module is able to adaptively generate a context-aware representation for each user from a large pool of concepts. pool to adaptively infer a sparse set of concepts for each user BERT4Rec [12]. This model employs a deep bi-directional self-attention mechanism to model sequences of user behavior.

- **Experimental environment**

All experiments were done in a T4 GPU environment on the Google Colab platform, with a hardware configuration including an NVIDIA T4 GPU and 16GB of video memory, a software environment Python version 3.9 and the PyTorch 2.0 deep learning framework, in addition to dependent libraries such as RecBole, NumPy, Pandas, and Matplotlib.

### Experimental results and analysis

- **Performance comparison with baseline**

On different datasets, we compared the performance with four baseline models, and the results are shown in Table 2. The comparison shows that the model based on the hybrid attention mechanism in the frequency and time domain outperforms the other baseline models in all evaluation metrics, especially in the Recall@10 and NDCG@10 metrics. This indicates that the joint consideration of frequency and time domain information can effectively improve the performance of course recommendation systems.

**Table 2:** Model performance comparison.

Dataset	Metric	CORE	LightSANs	SINE	BERT4Rec	Proposed Model	Improv.
MOOCCube	hit@10	0.4217	0.4528	0.3964	0.3628	**0.4534**	0.1%
	mrr@10	0.1219	0.1895	0.1437	0.1640	**0.2018**	6.4%
	ndcg@10	0.1924	0.2516	0.2031	0.2109	**0.2618**	4.0%
ml-100k	hit@10	0.1347	0.1262	0.0530	0.1389	**0.1474**	6.1%
	mrr@10	0.0459	0.0374	0.0164	0.0364	**0.0461**	0.4%
	ndcg@10	0.0617	0.0578	0.0248	**0.0674**	0.0664	-
Amazon_Books	hit@10	0.7037	0.7043	0.6087	0.4379	**0.0674**	1.0%
	mrr@10	0.3633	0.4628	0.4274	0.2310	**0.4757**	2.8%
	ndcg@10	0.4482	0.5207	0.4712	0.2815	**0.5305**	1.9%

Experimental results from three datasets demonstrate that the proposed model performs exceptionally well across most metrics, exhibiting particularly significant advantages on the representative datasets MOOCCube and Amazon_Books. On the MOOCCube dataset, although the Hit@10 metric shows only a marginal 0.1% improvement (0.4534 vs. 0.4528) over the optimal baseline model LightSANs, the model achieves significantly higher values on MRR@10 and NDCG@10 key indicators reflecting ranking quality reaching 0.2018 and 0.2618, respectively, representing significant improvements of 6.4% and 4.0% over LightSANs. This demonstrates that our model matches top models in precisely identifying users Top-10 needs, but its true strength lies in prioritizing more relevant and higher quality course recommendations.

On the ml-100k dataset, the model outperformed BERT4Rec by 6.1% in Hit@10 but slightly underperformed in NDCG@10. This indicates the model effectively uncovers users’ latent interests in this scenario, though ranking precision still holds optimization potential. Notably, the model demonstrates its strongest adaptability on the larger and more complex Amazon_Books dataset, outperforming the optimal baseline across all metrics. It achieves improvements of 1.0%, 2.8%, and 1.9% in Hit@10, MRR@10, and NDCG@10, respectively.

The proposed hybrid attention model demonstrates remarkable robustness in handling extremely sparse data (99.5% sparsity in MOOCCube), achieving state-of-the-art performance across all datasets. This success stems from the synergistic dual-pathway architecture: the temporal attention module effectively captures limited local dependencies within sparse sequences, while the frequency-domain component provides a global perspective to extract noise-resistant, steady-state interest patterns. This combination proves particularly advantageous for mild cold-start scenarios where minimal interaction data exists, as the frequency-domain analysis can infer stable user preferences from limited sequences. However, the model does not address absolute cold-start scenarios with zero interactions, as it lacks mechanisms to incorporate auxiliary information like user profiles or item content. This limitation, along with further enhancing the model’s adaptability to diverse data characteristics, represents a key direction for future research.

- **Ablation experiments**

In order to validate the contribution of the hybrid attention mechanism in the frequency and time domains in the model, this paper conducts ablation experiments in the dataset MOOCCube, including the three cases of using only the frequency domain, only the time domain, and a combination of the time and frequency domains, and the results of the experiments are shown in Table 3.

**Table 3:** Experimental results of ablation experiments.

Methods	mrr@10	ndcg@10	hit@10
# Time domain	0.1807	0.2384	0.4234
# Frequency domain	0.1780	0.2379	0.4316
# Time domain combined + Frequency domain	**0.2018**	**0.2618**	**0.4534**

The ablation experiments provide compelling evidence for the superiority of our dual-domain fusion approach. The full model, which integrates both temporal and frequency domains, achieves the best performance across all metrics (MRR@10: 0.2018, NDCG@10: 0.2618, Hit@10: 0.4534). More importantly, the performance gap between the full model and its single-domain variants quantitatively validates our core argument: domain fusion is not merely beneficial but essential for comprehensively understanding user behavior.

The most significant improvements were observed on NDCG@10 and Hit@10 metrics, where the full model outperformed single-domain approaches by approximately 10% and 5%, respectively. This specific pattern of results indicates that the key advantage of our approach lies in its enhanced ranking quality and Top-N recommendation accuracy. We attribute this superiority to the complementary roles of the two domains: the temporal domain module effectively captures local, sequence-dependent relationships in recent learning behavior, while the frequency domain component excels at identifying global, periodic interest patterns that are robust to sparse and noisy data. This synergistic combination enables the model to generate recommendations that are not only contextually relevant in the short term but also aligned with users’ stable, long-term learning habits, thereby effectively addressing the fundamental limitations of single-domain-dependent models.

- **Parameter sensitivity analysis**

In order to further validate the impact of key hyperparameters in the model on the recommendation performance, this paper conducts sensitivity experiments on global_ratio (frequency domain component ratio) and spatial_ratio (spatial domain to frequency domain ratio), which are conducted in in the dataset MOOCCube.

**(1) Global_ratio**

**Fig 2:**
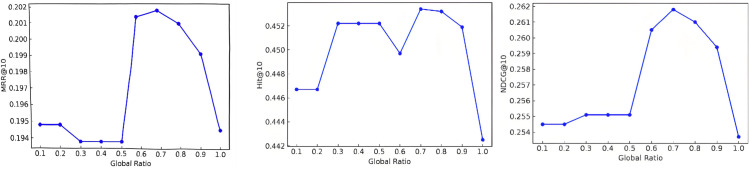
Results of mrr@10, hit@10 and ndcg@10 for different values of the global_ratio parameter.

The parameter global_ratio represents the proportion of frequency domain components introduced in the model, which is mainly used to control the contribution of frequency domain features to the overall recommendation effect of the model. The value of global_ratio is set to [0.1,0.2,0.3,0.4,0.5,0.6,0.7,0.8,0.9,1.0], and experiments are carried out on the MOOCCube dataset, with the evaluation metrics of mrr@10, hit@10 and ndcg@10, and the results are shown in Fig 2.

From the analysis of the experimental results in the above figure, mrr@10 (Mean Reciprocal Rank@10) fluctuates less under different global_ratio settings, with an optimal value of 0.2018, which occurs when global_ratio= 0.7. ndcg@10 (Normalized Discounted Cumulative Gain@10) shows an increasing and then decreasing trend with increasing global_ratio, with an optimal value of 0.2618, which occurs at global_ratio = 0.7. hit@10 (Hit Rate@10) shows a similar trend to ndcg@10 with different global_ratio settings, with an optimal value of 0.4534. The optimal value is 0.4534, which also occurs at global_ratio is 0.7.

From the experimental results, it can be seen that the appropriate setting of global_ratio is an important factor to improve the model performance. In particular, at global_ratio = 0.7, the model achieves the best performance on all metrics (mrr@10, ndcg@10 and hit@10). This suggests that a reasonable trade-off between global_ratio settings can help improve the relevance and coverage of recommendation results in recommender systems. However, when global_ratio is too large (e.g., close to 1), the recommendation performance degrades significantly because too high a weighting causes the model to ignore the contribution of other features.

**(2) Spatial_ratio**

**Fig 3:**
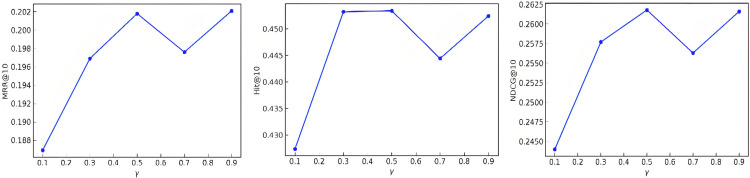
Results of mrr@10, ndcg@10 and hit@10 for different values of the spatial_ratio parameter.

The value of spatial_ratio (γ) represents the proportion of time domain and frequency domain features, the larger γ is, the more the model relies on time domain features. In this paper, the values of γ are [0.1, 0.3, 0.5, 0.7, 0.9], and the performance of the model on the metrics mrr@10, ndcg@10, and hit@10. The value of γ represents the weighting of the features in the time domain and the features in the frequency domain, and the larger γ is, the more the model relies on the features in the time domain. The experimental results are shown in Fig 3.

From the above figure, it can be seen that γ = 0.5 performs the best among all the metrics, with mrr@10, ndcg@10 and hit@10 reaching the peak in the dataset MOOCCube. This indicates that when the proportion of features in the time domain and frequency domain is balanced, the model can make full use of the complementary information of the two features, thus improving the recommendation effect.

## Conclusion

This study proposes a course recommendation model based on a hybrid frequency-time domain attention network, aiming to address the fundamental limitations of existing temporal recommendation methods that overly rely on single-time-domain analysis and struggle to comprehensively capture user behavior patterns. By introducing the Fast Fourier Transform to extract global frequency-domain features and combining it with a self-attention mechanism to capture local temporal dynamics, this model achieves dual-domain collaborative modeling of user learning behavior for the first time in the MOOC context. Experiments on public datasets demonstrate that the model significantly outperforms mainstream temporal models such as BERT4Rec and LightSANs across multiple key metrics, validating its superiority. Further ablation experiments demonstrate that dual-domain fusion yields approximately 10% improvement in NDCG@10 and 5% in Hit@10 performance. This empirically confirms that incorporating frequency-domain information effectively compensates for the shortcomings of purely temporal models, providing a more comprehensive perspective for user behavior representation.

Despite its promising performance, this study has certain limitations that point to valuable future research directions. The proposed model, in its current form, is primarily designed to learn from existing user interaction sequences and does not address the absolute cold-start problem for users with no historical data, as it lacks a mechanism to incorporate auxiliary information such as user profiles or course content. Furthermore, the model’s generalization capability and robustness across vastly different educational platforms and demographic contexts require further validation.

Building upon these limitations, our future work will focus on two promising paths: first, developing meta-learning or transfer learning frameworks to enhance the model’s cross-domain adaptability and its performance in data-scarce scenarios; second, exploring multimodal fusion techniques that integrate side information (e.g., knowledge graphs of course prerequisites, user demographics) into the hybrid attention architecture to effectively resolve the absolute cold-start challenge and enrich the understanding of user interests.
